# The Biosafety Research Road Map: The Search for Evidence to Support Practices in the Laboratory—SARS-CoV-2

**DOI:** 10.1089/apb.2022.0039

**Published:** 2023-06-05

**Authors:** Stuart D. Blacksell, Sandhya Dhawan, Marina Kusumoto, Kim Khanh Le, Kathrin Summermatter, Joseph O'Keefe, Joseph Kozlovac, Salama Suhail Almuhairi, Indrawati Sendow, Christina M. Scheel, Anthony Ahumibe, Zibusiso M. Masuku, Kazunobu Kojima, David R. Harper, Keith Hamilton

**Affiliations:** ^1^ Mahidol-Oxford Tropical Research Medicine Unit, Faculty of Tropical Medicine, Mahidol University, Bangkok, Thailand.; ^2^ Centre for Tropical Medicine and Global Health, Nuffield Department of Medicine, Nuffield Department of Medicine Research Building, University of Oxford, Oxford, United Kingdom.; ^3^ Institute for Infectious Diseases, University of Bern, Bern, Switzerland.; ^4^ Ministry for Primary Industries, Wellington, New Zealand.; ^5^ U.S. Department of Agriculture, Agricultural Research Service, Beltsville, Maryland, USA.; ^6^ Abu Dhabi Agriculture and Food Safety Authority, Abu Dhabi, United Arab Emirates.; ^7^ Research Center for Veterinary Science, National Research and Innovation Agency, Indonesia.; ^8^ WHO Collaborating Center for Biosafety and Biosecurity, Office of the Associate Director for Laboratory Science, Center for Global Health, Centers for Disease Control and Prevention, Atlanta, Georgia, USA.; ^9^ Nigeria Centre for Disease Control and Prevention, Abuja, Nigeria.; ^10^ National Institute for Communicable Diseases of the National Health Laboratory Services, Sandringham, South Africa.; ^11^ Department of Epidemic and Pandemic Preparedness and Prevention, World Health Organization, Geneva, Switzerland.; ^12^ The Royal Institute of International Affairs, London, United Kingdom.; ^13^ World Organisation for Animal Health (OIE), Paris, France.

**Keywords:** SARS-CoV-2, pathogen characteristics, biosafety evidence, biosafety knowledge gap, biorisk management

## Abstract

**Introduction:**

The SARS-CoV-2 virus emerged as a novel virus and is the causative agent of the COVID-19 pandemic. It spreads readily human-to-human through droplets and aerosols. The Biosafety Research Roadmap aims to support the application of laboratory biological risk management by providing an evidence base for biosafety measures. This involves assessing the current biorisk management evidence base, identifying research and capability gaps, and providing recommendations on how an evidence-based approach can support biosafety and biosecurity, including in low-resource settings.

**Methods:**

A literature search was conducted to identify potential gaps in biosafety and focused on five main sections, including the route of inoculation/modes of transmission, infectious dose, laboratory-acquired infections, containment releases, and disinfection and decontamination strategies.

**Results:**

There are many knowledge gaps related to biosafety and biosecurity due to the SARS-CoV-2 virus's novelty, including infectious dose between variants, personal protective equipment for personnel handling samples while performing rapid diagnostic tests, and laboratory-acquired infections. Detecting vulnerabilities in the biorisk assessment for each agent is essential to contribute to the improvement and development of laboratory biosafety in local and national systems.

## Introduction

The World Organization for Animal Health, World Health Organization (WHO), and Chatham House are currently collaborating to improve the sustainable implementation of laboratory biological risk management, particularly in low-resource settings. The Biosafety Research Roadmap project aims to support the application of laboratory biological risk management and improve laboratory sustainability by providing an evidence base for biosafety measures (including engineering controls) and evidence-based biosafety options for low-resource settings. This will inform strategic decisions on global health security and investments in laboratory systems. This study involves assessing the current evidence base required for implementing laboratory biological risk management, aiming to provide better access to evidence, identifying research and capability gaps that need to be addressed, and providing recommendations on how an evidence-based approach can support biosafety in low-resource settings.

In this study, we present the general characteristics of SARS-CoV-2, the current biosafety evidence, and available information regarding laboratory-acquired infections and laboratory releases.

## Materials and Methods

A 15 member technical working group (TWG) was formed to develop a Biosafety Research Roadmap (BRM) with the goal of supporting the application of laboratory biological risk management and improving laboratory sustainability by providing an evidence base for biosafety measures.

The TWG conducted a gap analysis for a selected list of priority pathogens on procedures related to diagnostic testing and associated research for those pathogens, including but not limited to sample processing, testing, animal models, tissue processing, necropsy, culture, storage, waste disposal and decontamination. To achieve this, the TWG screened databases, websites, publications, reviews, articles, and reference libraries for relevant data. The main research domains used to perform the literature searches were the ABSA database, Belgian Biosafety Server, US centers for disease, control and prevention (CDC) reports, WHO reports, PubMed, and internet searches for terms related to biosafety matters, including, for example, inactivation, decontamination, laboratory-acquired infections, laboratory releases and modes of transmission. The summary of evidence and potential gaps in biosafety was divided into five main sections: route of inoculation/modes of transmission, infectious dose, laboratory-acquired infections, containment releases, and disinfection and decontamination strategies.

## General Characteristics

The SARS-CoV-2 virus is the causative agent of the COVID-19 pandemic. It is a member of the family *Coronaviridae* and a single-stranded positive-sense RNA virus. SARS-CoV-2 is highly transmissible and more transmissible than SARS-CoV and Middle East respiratory syndrome, coronavirus^1^ with the R0 dependent on the variant (i.e., Alpha, Delta, and Omicron). The virus has been shown to experimentally infect cats, ferrets, hamsters, bats, and nonhuman primates. In contrast, natural (infected human-to-animal) infections have been noted in dogs, cats, and mink. There are cases of potential animal-to-human infection being investigated on mink farms.^2^ Animal models are in development for studying SARS-CoV-2 pathogenesis, transmission, treatment, and vaccine efficacy. Chu et al provided a brief comparison of the attributes of hamsters, wild-type and transgenic mice, ferrets, and nonhuman primates as models for SARS-CoV-2 research.^3^

Virus variants are identified based on mutations they acquire when passaged through human hosts, with many key mutations occurring in the spike protein. New variants and subvariants with changes in transmissibility and immune evasion characteristics continue to emerge at the time this article was submitted for publication. SARS-CoV-2 spike glycoprotein forms trimers on the surface of virions and is the main determinant for cell tropism.^4^ The spike protein consists of an S1 subunit, which binds to the host entry receptor, the angiotensin-converting enzyme 2,^5,6^ and the S2 subunit, which mediates membrane fusion. Infections starts in the upper airway and, if not cleared by the host immune response spreads to the lower respiratory tract, including cells in the lung.

Toward the end of 2019, SARS-CoV-2 virus caused an outbreak in Wuhan, China, which spread rapidly worldwide, resulting in a global pandemic.^7^ The virus is mainly spread through human-to-human transmission. Although most infections result in mild to moderate illness, it has an ∼1% fatality rate, with 3–20% of those infected requiring hospitalization.^8^ Of those hospitalized, 10–30% are placed in intensive care.^9^ Most infections range from asymptomatic to symptoms of mild to moderate respiratory disease, cough, fever, headache, myalgia, and diarrhea. Severe cases progress to shortness of breath due to low blood oxygen. They can progress to respiratory failure and death, and may also lead to extrapulmonary disease, including gastrointestinal symptoms and acute cardiac, kidney, and liver injury, in addition to cardiac arrhythmia, coagulopathy, and shock.^10^

The CDC estimates of the percentage of people in the U.S. infected with SARS-CoV-2 that develop “long-COVID,” where symptoms last 3 or more months after first contracting the virus range between 7.5% and 20%.^11^ In addition, multi-system inflammatory syndrome in children (MIS-C), a rare but severe complication associated with SARS-CoV-2 infection has been reported. It commonly presents as abdominal pain, vomiting, diarrhea, rash, conjunctivitis, and hypotension. and is indicative of inflammation across multiple body systems. There is still no conclusion as to exactly when the virus first infected humans; genetic evidence suggests the virus is a zoonotic agent that originated in animals.^12^

### Treatment and Prophylaxis

Prophylaxis for SARS-CoV-2 is through vaccination, 8 of which are WHO-approved (Moderna, Pfizer, Janssen, AstraZeneca, Covishield, Sinopharm, Covaxin, and Sinovac).^13^ Some treatments have shown benefits, but further research is required to confirm efficacy.^12^ Symptom management using over-the-counter antipyretics, analgesics, or antitussives for fever, headache, myalgias, and cough is recommended for nonhospitalized adults with mild to moderate symptoms. Several antiviral therapeutic options are available and recommended to reduce the risk of hospitalization or death in adults at high risk of progression to severe disease. However, as the virus mutates, monoclonal antibody therapies have become less effective or ineffective as pre-exposure prophylaxis and treatment measures against omicron subvariants.

Updates to treatment recommendations are provided by the National Institutes of Health COVID-19 Treatment Guideline Panel and change periodically based on the emergence of variants and subvariants ability to evade treatment regimens.^14^ The antiviral drug Remdesivir is approved by the U.S. Food and Drug Administration, and Ritonavir-boosted nirmatrelvir (Paxlovid) and molnupiravir have received Emergency Use Authorizations for the treatment of COVID-19. Paxlovid is recommended by WHO for patients with mild and moderate symptoms at the highest risk of hospital admission.

### Diagnostics

Early diagnosis is essential for disease management and control. The current gold standard for SARS-CoV-2 diagnosis is molecular detection, specifically Nucleic Acid Amplification Tests (NAATs) such as reverse transcription—polymerase chain reaction (RT-PCR).^12,15^ NAATs are highly sensitive and highly specific tests that detect one or more viral RNA genes. Although NAATs are indicative of a current infection because pieces of viral RNA may stay in a person's body for up to 90 days after individual tests positive, NAATs should not be used to test someone who has tested positive in the past 90 days. Other detection methods, such as antigen tests, have complimented the molecular diagnosis.^15^

Antigen tests are immunoassays that detect a specific viral antigen. There has been a proliferation in the development of antigen tests, with many sold for at-home, point-of-care, test location, and laboratory use. Because they are less sensitive than NAAT, a negative result using an antigen test does not mean infection with SARS-CoV-2 virus can be ruled out, and repeat testing (after 48 h) or NAAT has been recommended.^16^ Antigen tests that have been vetted and approved by governmental agencies often have similar specificity but less sensitive as compared with NAATs. Two other tests have been developed, including Diagnostic Breath Tests for volatile organic compounds associated with COVID-19 disease and Genotyping Tests such as Phylogenetic Assignment of Named Global Outbreak that differentiates virus lineages and/or identifies specific SARS-CoV-2 mutations. These latter two types of tests are conducted in hospital diagnostic laboratories certified to conduct high-complexity tests.

## Biosafety Evidence

Despite the relatively short time that the virus has been circulating in the broader community, enormous international efforts have been made to understand its virological characteristics and safety strategies for preventing exposure and infection.

### Modes of Transmission

The primary mode of transmission is droplet and aerosol inhalation (person-to-person transfer), followed by contact with contaminated fomites and surfaces. In a minority of cases perinatal transmission occurs with infected women in the third trimester of pregnancy.^17,18^ Regarding aerosols, van Doremalen et al report, “The half-lives of SARS-CoV-2 and SARS-CoV-1 were similar, with median estimates of approximately 1.1 to 1.2 hours…aerosol transmission of SARS-CoV-2 is plausible.”^19^

Studies into the persistence of viable virus on fomites indicated that “The longest viability of both viruses was on stainless steel and plastic; the estimated median half-life of SARS-CoV-2 was approximately 5.6 hours on stainless steel and 6.8 hours on plastic…fomite transmission of SARS-CoV-2 is plausible.”^19^ In the context of infection control and surface decontamination in the laboratory setting, it is important to note that the aforementioned data describe the half-life of SARS-CoV-2. The authors presented additional data demonstrating the titers of viable virus that could be recovered for significantly longer periods, in some cases days, from some inoculated test materials.

### Infectious Dose

Ten 50% tissue culture infectious doses (TCID_50_) of SARS-CoV-2/human/GBR/484861/2020, a D614G-containing pre-alpha wild-type virus delivered intranasally was shown to infect 50% of healthy volunteers in a human challenge study.^20^

### Laboratory-Acquired Infection

Only one SARS-CoV-2 laboratory-acquired infection (LAI) has been reported to date. Academia Sinica in Taiwan reported that on December 11, 2021, an assistant researcher, case No. 16,816, had contracted COVID while working in a Biosafety Level 3 (BSL-3) facility. The laboratory is located inside Academia Sinica's Genomics Research Center in Taipei's Nangang District.^21^

### Disinfection and Decontamination

The SARS-CoV-2 virus is susceptible to various chemical and physical inactivation procedures.

#### Chemical

Lysis buffer is commonly used in extraction kits for RT-PCR diagnostics, such as QIAGEN's ATL Buffer (1–10% sodium dodecyl sulfate [SDS]) and VXL Buffer (30–50% guanidine hydrochloride, 1–10% t-octylphenoxypolyethoxyethanol [TritonX-100]). Both ATL and VXL buffers reportedly reduce SARS-CoV-2 infectivity by at least 6 log_10_, as no cytopathic effect was observed for all replicates.^22^ Detergents often used in nucleic acid extractions, such as 0.5% SDS, 0.5% Triton X-100, 0.5% NP-40, and nucleic acid extraction reagents, Trizol, or Trizol LS, are effective for SARS-CoV-2 inactivation; however, Tween 20 alone did not inactivate SARS-CoV-2 under the same conditions for serological assays.^23,24^

Conventional chemical disinfectants, glutaraldehyde (0.5–2%), formaldehyde (0.7–1%), and povidone-iodine (0.1–0.75%), readily inactivate coronaviruses.^25,26^ Sodium hypochlorite (0.1%) efficiently inactivates SARS-CoV-2 at different concentrations within 1 min.^26^ WHO recommends 1% sodium hypochlorite.^26^ Available chlorine of 250, 500, and 1000 mg/L required 20, 5, and 0.5 min to inactivate SARS-CoV-2, respectively.^25^

WHO recommends 62–71% ethanol solution to inactivate SARS-CoV-2.^26^ Ethanol at 30% concentration for 1 min and 40% and above for 0.5 min, ethanol efficiently inactivates SARS-CoV-2.^25^ It has been reported that 70% and 80% 2-propanol efficiently inactivated coronaviruses at different concentrations ≤1 min.^26^ A 600-fold dilution of 17% concentration of di-N-decyl dimethyl ammonium bromide (283 mg/L) and the same concentration of di-N-decyl dimethyl ammonium chloride required only 0.5 min to inactivate SARS-CoV-2.^25^ For gaseous decontamination purposes, 8700 ppm hypochlorous acid vapor and 56,400 ppm hydrogen peroxide vapor effectively reduce or inactivate SARS-CoV-2.^27^

#### Thermal

Thermal methods have been widely used for the inactivation of samples before SARS-CoV-2 diagnostic testing, especially molecular-based diagnostics. Heat takes ∼30 min at 56°C, 10 min above 70°C, or 5 min above 90°C to inactivate the SARS-CoV-2.^25^ Several studies have reported the effectiveness of heating diagnostic samples to inactivate SARS-CoV-2 at 56°C for 30 min or 65°C for 15 min^28^ or 30 min^29^ while maintaining genomic stability for NAAT assays. Complete sterilization of respirators intentionally contaminated with 6 Logs SARS-CoV-2 virus was achieved by autoclaving at 121°C for 15 min (40 min autoclave cycle).^30^ Autoclave cycles must be validated for different types and sizes of loads.

#### Fumigation

Complete sterilization of respirators intentionally contaminated with 6 Logs SARS-CoV-2 virus was achieved using vaporized hydrogen peroxide with a peak exposure of 750 ppm achieved during 3 min conditioning, 2 h decontamination, and 2 h dwell time phases.^30^ The study was conducted in a small glovebox. If a spill were to occur in a room, or the objective was to decontaminate a large space, the vaporised hydrogen peroxide cycle would have to be validated to ensure decontamination is successful.

#### Radiation

Ultraviolet-C (UVC) wavelengths </ = 280 nm effectively inactivates SARS-CoV-2.^31^ Inactivation by UVC radiation at 254 nm has shown to be effective at a minimum ultraviolent energy of 0.04 J/cm^2^.^23^ However, it is important to note that UV light bulbs require frequent replacement and monitoring for bactericidal activity and the absence of shadows. As such, they would not be recommended as a primary means of disinfection.

A complete list of the evidence is provided in Table 1.

**Table 1.** Detailed pathogen biosafety evidence for SARS-CoV-2

Method	Details	Evidence (direct quote where available)	Reference	Evidence gap? (yes/no)
Modes of transmission	Aerosol	“…the half-lives of SARS-CoV-2 and SARS-CoV-1 were similar in aerosols, with median estimates of approximately 1.1 to 1.2 hours…aerosol transmission of SARS-CoV-2 is plausible”	^19^	No
	Fomite infection	“The longest viability of both viruses was on stainless steel and plastic; the estimated median half-life of SARS-CoV-2 was approximately 5.6 hours on stainless steel and 6.8 hours on plastic…fomite transmission of SARS-CoV-2 is plausible”	^19^	
	Droplet	“The COVID-19-RdRp/Hel assay was significantly more sensitive than the RdRp-P2 assay for the detection of SARS-CoV-2 RNA in nasopharyngeal aspirates/swabs or throat swabs (*P* = 0.043), saliva (*P* < 0.001)”^1^	^36^	
		“All cases were first tested when symptoms were still mild or in the prodromal stage…Diagnostic testing suggests that simple throat swabs will provide sufficient sensitivity at this stage of infection”^2^	^37^	
		“High viral loads of severe acute respiratory syndrome coronavirus 2 (SARS-CoV-2) have been detected in oral fluids of coronavirus disease 2019 (COVID-19) −positive patients (6), including asymptomatic ones (7)”	^38^	
Infectious dose	Human trial with pre-alpha variant	“With a low inoculum dose of 10 TCID_50_, robust viral replication was observed in 53% of sero- negative participants. After an incubation period of less than 2 days, VLs rose rapidly, peaking at high levels with infectious virus production for over 1 week. Symptoms were present in 89% of infected individuals but, despite high VLs, were consistently mild to moderate, transient and predominantly confined to the upper respiratory tract”	^20^	No
	All other variants and subvariants	No evidence for infectious dose in humans		Yes
LAIs	1 LAI report	Only one COVID LAI has been reported to date. Academia Sinica in Taiwan reported on December 11, 2021 an assistant researcher, case No. 16,816, had contracted COVID while working in a P3 (Biosafety Level 3) facility. The laboratory is located inside Academia Sinica's Genomics Research Center (GRC), which is situated in Taipei's Nangang District.	^21^	No
Animal models	Optimized and defined research models	Selection of the optimal model depends on which questions the research will address. Currently, multiple models may have to be used to generate definite conclusions to specific questions	^4^	Yes
Fumigation	Vaporized hydrogen peroxide	Sterilization of respirators intentionally contaminated with 6 Logs SARS-CoV-2 virus was achieved using vaporized hydrogen peroxide with a peak exposure of 750 ppm achieved during 3 min conditioning, 2 h decontamination, and 2 h dwell time phases	^30^	No
Chemical inactivation	Detergent SDS—Detergent/Virus Ratio: 0.1%, 0.5%—Contact time: 30 min Trixton X-100—Detergent/Virus Ratio: 0.1%—Contact time: 30 min	“Our detergent inactivation data, indicating that SDS, Triton X-100 and NP-40, but not Tween 20, can effectively inactivate SARS-CoV-2 both in tissue culture fluid, and also in pooled NP and OP swab fluid”	^24^	No
	Detergent/Virus Ratio: 0.5% Contact time: <2 min NP-40—Detergent/Virus Ratio: 0.1%, 0.5%—Contact time: 30 min Trizol/Trizol LS (Details not stated)	“We successfully demonstrated this with five different compounds: 0.5% SDS, 0.5% Triton X-100, 0.5% Nonidet P40, Trizol, and Trizol LS. Conversely, Tween 20 did not inactivate SARS-CoV-2 under the same conditions”	^23^	
	Lysis Buffer ATL Buffer (1–10% SDS) VXL Buffer (30–50% guanidine hydrochloride, 1–10% t-octylphenoxypolyethoxyethanol [TritonX-100])	“ATL and VXL buffers were able to reduce infectivity by at least 6 log10, as no CPE was observed for all replicates… ATL or VXL should be preferred to AVL”	^22^	
	Glutaraldehyde (0.5–2%), Formaldehyde (0.7–1%), Povidone-iodine (0.1–0.75%) 1% sodium hypochlorite or an 62–71% ethanol solution	“Additionally, glutaraldehyde (0.5–2%), formaldehyde (0.7–1%), and povidone-iodine (0.1–0.75%) could readily inactivate coronaviruses. WHO recommends 1% sodium hypochlorite or an 62–71% ethanol solution”	^26^	
	BD Max buffer (Becton, Dickinson), Cobas^®^ lysis buffer (Roche, DE), Cobas^®^ viral transport medium (Roche), NucliSENS^®^ EMAG^®^ lysis buffer (bioMérieux), Maxwell lysis buffer (Promega), Panther Fusion™ lysis buffer (Hologic^®^), and Sun-Trine^®^ viral transport medium (SunTrine^®^ Biotechnologies)	“All commercial buffers tested herein, therefore, yield a >6 log_10_ reduction of active SARS-CoV-2 replication”	^39^	
	SDS at final concentrations of 2.0%, 1.0%, and 0.5% for 30 and 10 min	“All SDS concentrations tested, therefore, yield a >6 log_10_ reduction of active virus replication”	^39^	
	Chlorine 250 mg/L for 20 min Chlorine 500 mg/L for 5 min Chlorine 1000 mg/L for 0.5 min	“Available chlorine of 250 mg/L, 500 mg/L, and 1000 mg/L required 20 min, 5 min, and 0.5 min to inactivate SARS-CoV-2, respectively”	^25^	
	Ethanol 30% for 1 min and Ethanol 40% and above for 0.5 min	“Ethanol at 30% concentration for 1 min and 40% and above for 0.5 min, ethanol efficiently inactivates SARS-CoV-2”	^25^	
	283 mg/L di-N-decyl dimethyl ammonium bromide 283 mg di-N-decyl dimethyl ammonium chloride	A 600-fold dilution of 17% concentration of di-N-decyl dimethyl ammonium bromide (283 mg/L) and the same concentration of di-N-decyl dimethyl ammonium chloride required only 0.5 min to inactivate the virus efficiently.	^25^	
	8,700 ppm hypochlorous acid (vapor) 56,400 ppm hydrogen peroxide (vapor)	“The result obtained revealed that 8,700 ppm hypochlorous acid solution in the form of dry fog was required to inactivate SARS-CoV-2 under our experimental condition” “Moreover, 56,400 ppm hydrogen peroxide solution reduced the infectious titer of SARS-CoV-2”	^27^	
Thermal inactivation	56°C for 30 min	“Heat inactivation at 56 degrees C for 30 min did not affect the qualitative rRT-PCR detection of SARS-CoV-2”	^29^	No
	121°C for 15 min	Autoclaving respirators inoculated with 6 logs SARS-CoV-2 results in sterilization.	^30^	
	60°C for 1 h 56°C for 30 min 60°C for 30 min 56°C for 10 min 60°C for 10 min	“All heat protocols therefore also yield a >6 log_10_ reduction of active SARS-CoV-2 replication”	^39^	
	56°C for 30 min 65°C for 15 min	“In this study, we found that the SARS-CoV-2 is efficiently inactivated following incubation at 56°C or 65°C for 30 min or 15 min, respectively”	^28^	
	80°C for 1 h	“In this study, SARS-CoV-2 was successfully inactivated with a temperature of 80°C. Lower temperatures used to inactivate SARS-CoV showed that 56°C is only effective in the absence of fetal calf serum and temperatures up to 75°C are needed for successful inactivation of infected clinical samples”	^23^	
	30 min at 56°C 10 min above 70°C 5 min above 90°C	“Heat takes approximately 30 min at 56°C, 10 min above 70°C, or 5 min above 90°C to inactivate the virus”	^25^	
Radiation inactivation	UV radiation—0.04 J/cm^2^	“…we have demonstrated a method by which −2 can be rendered non-infectious through application of UV energy >0.04 J/cm^2^”	^23^	No
	UV radiation—0.2 to 140 J/cm^2^	“0.2 to 140 J/cm^2^”	^26^	
	UVC wavelengths </ = 280 nm	“UVC wavelengths (</ = 280 nm) were most effective for inactivating SARS-CoV-2, although inactivation rates were dependent on sample type. Results from this study suggest that UV radiation can effectively inactivate SARS-CoV-2 in liquids and dried droplets…”	^31^	

*Note:* This information is not exhaustive as the area is expanding rapidly. This information is correct as of November 2022.

LAIs, laboratory-acquired infections; SDS, sodium dodecyl sulfate; UV, ultraviolet; UVC, ultraviolet-C.

## Knowledge Gaps

### Infectious dose

The variations in infectious dose by variant type are still unknown. This may be a significant challenge given the number of circulating variants, and it may be expected that the infectious dose is different for each variant and route of transmission.

### Animal Models

Several animal models have been developed to study SARS-CoV-2 infection, but the optimal model selection depends on which questions the research will address. Currently, multiple models may have to be used to generate definite conclusions to questions. Identifying or optimizing models that address different research questions is critically needed.^32^

### Personal Protective Equipment When Using Rapid Diagnostic Tests

Since the middle of the pandemic, there has been a high volume of rapid tests globally, given the urgency to diagnose infection. The risk of infection to the laboratory worker or diagnostician when using rapid antigen tests or when preparing samples and loading assays that utilize cartridges on the open bench, such as GeneXpert cartridges, remains uncertain.^33^ Current WHO biosafety guidelines indicate that N95 respirators and biosafety cabinets are not necessarily required for this activity.^34^ However, it is strongly recommended that a site-specific risk assessment be conducted based on the agent and specific activities/operations being performed when specifying containment equipment and personal protective equipment (PPE).^34^

### Laboratory-Acquired Infections

There is presently only one LAI reported to date.^21^ However, it should be noted that until December 2021, no cases had been reported^.^^35^ Given a large number of community transmissions, it may be difficult to ascertain LAI cases, and it may be challenging to discriminate between community infection and LAIs. Although numerous studies have been conducted showing the increased prevalence in infection among healthcare workers who work with patients as compared with the general public, there are no similar data for individuals in diagnostic laboratories handling and analyzing patient samples. This information would inform LAI risks in laboratory diagnostic settings. The lack of mandatory laboratory-acquired infection reporting requirements in most countries further complicates this.

## Conclusions

As SARS-CoV-2 variants continue to evolve, it is important to understand how and which mutations specifically effect transmissibility, pathogenicity, and immune evasion. In addition, developing “pan-coronavirus” vaccines and therapeutic modalities is a priority. Each of these requires optimized and defined animal models that best answer the research question and provide relevance regarding human infection, infection prevention, and treatment.

Various chemical and physical inactivation processes have been shown to successfully inactivate the virus. Low-cost decontaminants include bleach and alcohol dilutions. The efficacy of these decontaminants regarding concentration and contact time are influenced by the type of material being decontaminated, bioburden and other factors and should be validated.

Although only one SARS-CoV-2 LAI has been reported to date, the state of the global pandemic calls for stringent biosafety measures to be exercised to minimize the risk of laboratory-acquired infections with SARS-CoV-2. All nonpropagative testing of specimens (sample processing, analysis of inactivated specimens, sequencing work, NAATs, etc.) can be conducted in a facility using heightened control measures in BSL-2 laboratories. In contrast, propagative work (handling of specimens with high titers of live virus, working with large quantities of virus, culturing, viral isolation, etc.) should be undertaken in a BSL-3 containment laboratory with inward directional airflow, employing heightened control measures, and consideration of enhanced PPE (i.e., disposable laboratory coat or sleeves, respiratory protection, and double gloves). An occupational health program should be in place to ensure individuals working with the virus are offered vaccines and testing and therapeutics are available.
